# A collection of genetically engineered *Populus* trees reveals wood biomass traits that predict glucose yield from enzymatic hydrolysis

**DOI:** 10.1038/s41598-017-16013-0

**Published:** 2017-11-17

**Authors:** Sacha Escamez, Madhavi Latha Gandla, Marta Derba-Maceluch, Sven-Olof Lundqvist, Ewa J. Mellerowicz, Leif J. Jönsson, Hannele Tuominen

**Affiliations:** 1 Department of Plant Physiology, Umeå University, Umeå Plant Science Centre (UPSC), SE-901 87 Umeå, Sweden; 2 Department of Chemistry, Umeå University, SE-901 87 Umeå, Sweden; 3 Department of Forest Genetics and Plant Physiology, Swedish University of Agricultural Sciences, Umeå Plant Science Centre (UPSC), SE-901 83 Umeå, Sweden; 4 INNVENTIA AB, RISE Bioeconomy, Drottning Kristinas väg 61 B, SE-114 28 Stockholm, Sweden

## Abstract

Wood represents a promising source of sugars to produce bio-based renewables, including biofuels. However, breaking down lignocellulose requires costly pretreatments because lignocellulose is recalcitrant to enzymatic saccharification. Increasing saccharification potential would greatly contribute to make wood a competitive alternative to petroleum, but this requires improving wood properties. To identify wood biomass traits associated with saccharification, we analyzed a total of 65 traits related to wood chemistry, anatomy and structure, biomass production and saccharification in 40 genetically engineered *Populus* tree lines. These lines exhibited broad variation in quantitative traits, allowing for multivariate analyses and mathematical modeling. Modeling revealed that seven wood biomass traits associated in a predictive manner with saccharification of glucose after pretreatment. Four of these seven traits were also negatively associated with biomass production, suggesting a trade-off between saccharification potential and total biomass, which has previously been observed to offset the overall sugar yield from whole trees. We therefore estimated the “total-wood glucose yield” (TWG) from whole trees and found 22 biomass traits predictive of TWG after pretreatment. Both saccharification and TWG were associated with low abundant, often overlooked matrix polysaccharides such as arabinose and rhamnose which possibly represent new markers for improved *Populus* feedstocks.

## Introduction

Sugars extracted from wood biomass represent a promising source of renewable biofuels and other green chemicals to sustainably replace petroleum-based products^1–4^. In particular, the biochemical conversion of lignocellulosic biomass holds great potential^3^, although improvements are needed at every step of the process^3^, starting with the feedstocks.

Tree species from the *Populus* genus represent interesting lignocellulosic feedstocks because they exhibit rapid growth even on marginal lands and are widely and efficiently cultivated^5,6^. Furthermore, the genomes of several *Populus* species have been sequenced^5,6^. Research efforts have focused on improving the biomass production of *Populus* feedstocks^7–10^. However, for biochemical conversion it is important to also consider woody biomass recalcitrance to enzymatic saccharification, requiring harsh pretreatments and therefore higher costs in industrial processes^11^.

Biomass recalcitrance has been studied in natural variants of the *Populus* genus^12–14^, showing that lignin amount and composition affect saccharification^14^, and revealing parts of the genetic relationships underlying lignin properties and other biomass traits, as well as their impact on wood recalcitrance^12,13^. Parallel approaches have relied on targeted genetic engineering of xylem cell walls, resulting in trees less recalcitrant to enzymatic saccharification, although sometimes at the expense of growth^15–22^. In particular, saccharification or the subsequent sugar conversion could be improved by genetic engineering altering the composition of matrix polysaccharides^16,17^, reducing the amount of lignin^18^ or modifying lignin composition^20,21^. Together, these studies provide useful information for future breeding or genetic engineering programs as well as a source for new, improved feedstocks. However, translating these tools and knowledge into practice requires further research into aspects such as trade-off between the reduction of recalcitrance and biomass production.

The present study contributes to bridging this knowledge gap by characterizing the relationship between biomass traits and susceptibility to enzymatic saccharification in a population of transgenic hybrid aspen (*Populus tremula* x *tremuloides*; hereafter *Populus*) known as the BioImprove collection. We estimated the glucose yield after pretreatment and 72 h enzymatic hydrolysis from the total wood biomass of each tree to identify diagnostic traits for the creation and selection of not only less recalcitrant but overall superior trees with increased sugar yield. Such selection could be applied in current breeding programs to enhance biochemical conversion rates. Furthermore, our collection of transgenic trees theoretically comprises combinations of traits that are not currently found in nature, paving the way for a deeper biological understanding of woody biomass and of the ways to improve it.

## Results

### The BioImprove *Populus* collection provides a trait library for characterizing wood biomass properties and glucose yield

**Figure 1:**
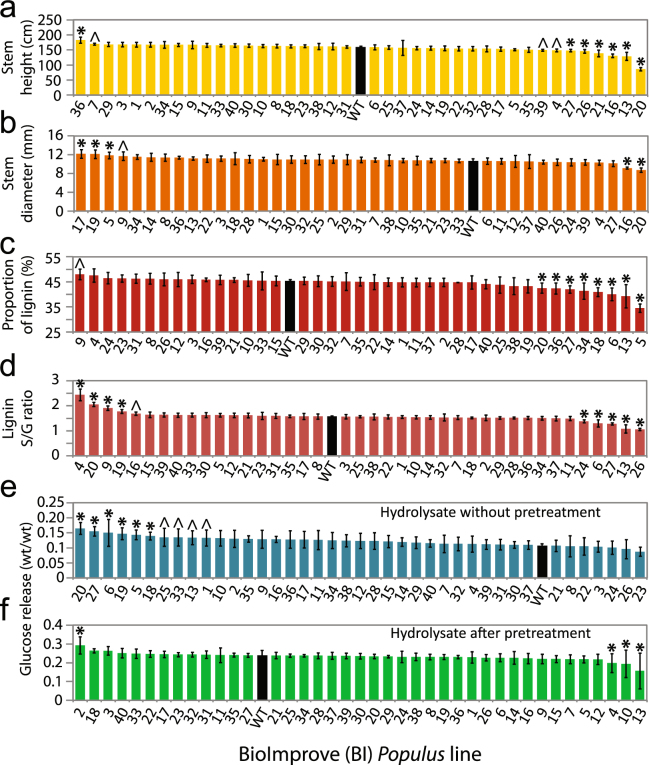
The BioImprove *Populus* collection provides a wide variation in major traits. (**a**,**b**) Growth-related traits: stem height (**a**) and stem diameter (**b**). (**c**,**d**) Biomass recalcitrance-related traits: proportion of lignin within the detected pyrolysate from biomass (**c**) and ratio of S- to G-units within the lignin polymer (**d**). (**e**,**f**) Saccharification-related traits: glucose release after a 72 h enzymatic hydrolysis without (**e**) or after (**f**) pretreatment. Histograms represent the average value for transgenic lines (color) and wild type (black). Error bars represent standard deviation. * and ^ indicate statistically significant differences from wild type (p < 0.05 and p < 0.1 respectively) following a post-ANOVA Fisher’s test (n = 3–5).

We investigated the relationships between wood traits and the potential of woody biomass for enzymatic saccharification in *Populus* trees by altering the expression of genes putatively regulating wood biomass properties. For this purpose, we utilized a collection of 40 transgenic *Populus* lines whose genetic modifications aimed at modifying the expression of 39 different genes (Dataset S1). These lines, as well as the wild-type T89 clone, were analyzed for three growth-related traits, 20 cell wall chemistry traits, 20 wood anatomy and structural traits and 22 saccharification traits (Dataset S2), thus generating a broad wood-related trait library. Notably, a wide variation was observed for major growth traits such as height and diameter (Fig. 1a,b), for traits critical for biomass recalcitrance such as lignin content and lignin monomer composition (Fig. 1c,d) and for analytical saccharification traits such as glucose release after 72 h of enzymatic hydrolysis without pretreatment or after a severe acidic pretreatment (Fig. 1e,f). This variation in quantitative traits between lines is valuable as it allows us to decipher how wood properties influence traits of interest, such as glucose yield.

**Figure 2:**
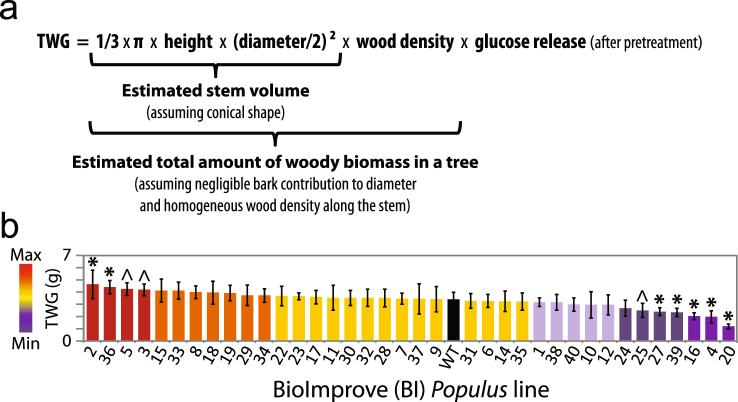
The BioImprove lines display a range of total-wood glucose yield (TWG). (**a**) Formula for estimation of a tree’s total-wood glucose yield after pretreatment and 72 h enzymatic hydrolysis, assuming conical shape, negligible bark contribution to diameter and homogeneous wood density. (**b**) TWG of the BioImprove *Populus* lines. Each histogram represents the average value for a transgenic *Populus* line (color) or wild type (black). Error bars represent standard deviation. * and ^ indicate statistically significant differences from wild-type (p < 0.05 and p < 0.1 respectively) following a post-ANOVA Fisher’s test (n = 3–5).

Notably, saccharification is usually expressed as the relative amount of sugar released per unit of biomass, which reflects the recalcitrance rather than the sugar yield of an entire tree. Trees with high saccharification may concomitantly suffer from growth defects, which may nullify the *in fine* sugar yield. Instead, ideal trees for biochemical conversion of biomass should combine high saccharification with sufficient growth to ensure superior yield from their total wood biomass. Therefore, we created a combinatorial trait – a tree’s “total-wood glucose yield”, which represents glucose yield after enzymatic saccharification either after acidic pretreatment (TWG; Fig. 2a; Dataset S2) or without pretreatment (TWGnp; Fig. S1a; Dataset S2). Our focus on glucose yield is justified by the fact that glucose is the most prominent product of saccharification. Interestingly, several BioImprove *Populus* lines exhibited significantly different TWG and TWGnp compared with the wild-type trees, and several lines outperformed the wild type both without and after pretreatment (Fig. 2b; Fig. S1b). Both the glucose released from saccharification without pretreatment and the corresponding TWGnp yielded only about half of what could be obtained after the severe acidic pretreatment condition (Fig. 2b; Fig. S1b; Dataset S2). Although we analyzed both conditions, we will place more emphasis on the pretreated samples which are more relevant to potential applications.

To identify variation in traits that could separate the lines based on TWG, we first performed a principal component analysis (PCA). The resultant PCA model displayed nine significant principal components (PCs; Dataset S3) explaining 78.6% of the variation in the data: 30.2% were explained by the two first PCs. Neither of the two first PCs (Fig. S2) nor any other combination of PCs (Dataset S3) could separate the lines based on their TWG. Hence, the variation in TWG was not associated with the main biological variation separating the lines in the PCA, implying the need for a different method to identify biomass properties associated with TWG.

### Certain traits are associated with total-wood glucose yield

**Figure 3:**
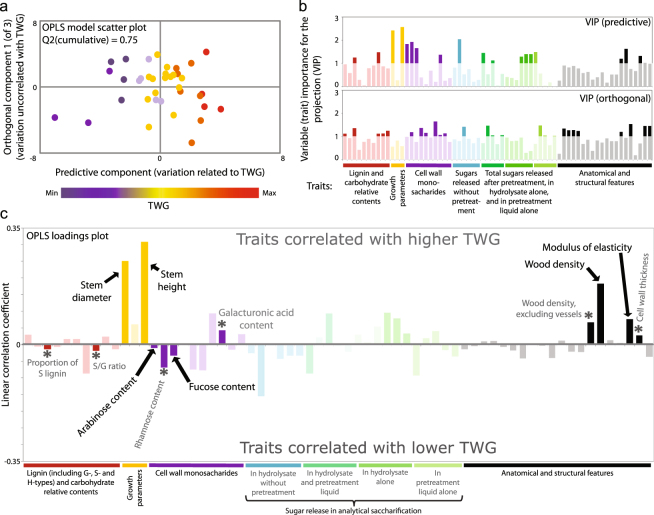
Certain traits contribute more than others to predicting TWG. (**a**) OPLS scatter plot showing the separation of the *Populus* lines (dots) horizontally along the predictive component for total-wood glucose yield (TWG). Vertical separation indicates variation not correlated with TWG. The lines were coloured by TWG. (**b**) Plots showing the variable importance for the projection (VIP) value for each trait for the predictive part of the model (up) and for the orthogonal part of the model (down). VIP values over 1 indicate important traits. (**c**) Contribution of each trait to the OPLS model. Apart from saccharification traits, traits with a VIP value over 1 for the predictive part of the model were emphasized by black text and arrows. Traits marked by (*) and annotated in grey are important (VIP value over 1) for both the predictive and the orthogonal part of the model. Q^2^ scores over 0.5 indicate significant predictivity of a model.

To overcome the limitations of the PCA analysis, we compared the 38 *Populus* lines whose TWG could be calculated (Fig. 2b; Dataset S2) using a supervised, predictive multivariate analysis. Orthogonal projection of latent structures (OPLS^23^) enables us to distinguish the variation related to a variable of interest, for instance TWG, from the unrelated (orthogonal) systematic variation. An OPLS model relying on all of the 65 recorded traits was generated which could separate the *Populus* lines with respect to TWG (Fig. 3a) in a significantly predictive manner (Q^2^ = 0.75).

In order to identify the traits that contribute most to predicting TWG in the BioImprove collection, we calculated each trait’s VIP (variable importance for the projection) values for both the TWG-predictive and TWG-orthogonal parts of the OPLS model (Fig. 3b; Dataset S3). Attempts to use VIP in order to reduce the number of traits used to predict TWG also strongly reduced the model’s predictivity. Although the model relied on all 65 traits, VIP values (Fig. 3b) indicated that some traits contributed more to predicting TWG than others. We therefore relied on significantly high VIP values (VIP > 1; Fig. 3c) to identify traits which appeared important for TWG prediction. We preferred to focus on predictive traits that are easily measurable, and hence applicable in current forest tree breeding practices. Therefore, the saccharification traits which are difficult to measure were not considered as feasible traits for TWG prediction. Among all the other traits, 12 traits were significantly associated with TWG in the OPLS model (Fig. 3c). Height, diameter and wood density were positively associated with TWG (Fig. 3c), as expected on the basis of the fact that TWG is a composite feature which integrates these traits. Consistent with the contribution of density to TWG, increased wood stiffness (modulus of elasticity) and cell wall thickness were also associated with higher TWG (Fig. 3c). Interestingly, galacturonic acid content was positively associated with TWG while arabinose, rhamnose and fucose contents were negatively associated with TWG (Fig. 3c), showing that quantitatively minor cell wall compounds could influence TWG under our pretreatment condition. Increases in S-type lignin content and in the ratio of S- to G-type lignin were weakly but significantly negatively associated with TWG in the OPLS model.

### Mathematical modeling predicts TWG, saccharification and biomass production from a subset of traits

The OPLS analysis revealed the possibility of predicting TWG from wood biomass traits in our dataset. However, our OPLS model relies on all traits, making it informative but difficult to apply to predict TWG from future datasets. Hence, we attempted to generate a mathematical model to predict TWG from only a subset of wood biomass traits. Such a model relying on a limited set of traits, especially traits which are easier to measure than saccharification, could indeed be used with future datasets to verify the general applicability of the model and to serve potential future applications.

**Figure 4:**
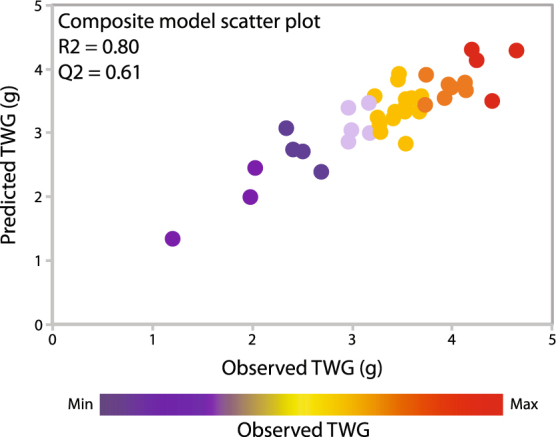
TWG can be predicted by a specific subset of traits in a composite model. Scatter plot showing for each *Populus* line (dots) the observed total-wood glucose yield (TWG, x-axis) versus the predicted TWG (y-axis). Q^2^ scores over 0.5 indicate significant predictivity of a model.

First, distinct individual models were generated for each of the four traits, height, diameter, wood density and glucose release after acidic pretreatment, from which TWG is calculated (Dataset S4). Then, by replacing each term in the TWG equation (Fig. 2a) with the corresponding model, we obtained a composite model to predict TWG (Dataset S4). In that way, the potential effect of predictive traits on TWG could be traced down to an effect on saccharification, on biomass production, or even both. The resulting composite model could predict TWG (Fig. 4) with significant accuracy (Q^2^ = 0.61). In contrast to the OPLS model, which included all the traits in the dataset, our composite mathematical model relied solely on 22 biomass traits (Table 1). Following the same procedure, another composite model was generated that could predict total-wood glucose yield without pretreatment (TWGnp; Fig. S2c) from a subset of 19 traits, with significant prediction accuracy (Q^2^ = 0.64). Any attempt to reduce the number of traits used in either of the composite models also greatly reduced the models’ predictivity, suggesting that TWG and TWGnp are complex traits emerging from intricate biological interactions.

**Table 1:** Wood traits predicting TWG* in the composite model.

Traits (contributing to either of the individual models, hence to the composite model)	Impact of the trait on TWG in the composite model
Proportion of S lignin	Positive
Ratio of S-type to G-type lignin	Negative**
Arabinose content	Negative**
Rhamnose content	Negative**
Fucose content	Negative
Modulus of elasticity (stiffness)	Positive**
Cell wall thickness	Positive**
Xylose content	Negative
Mannose Content	Negative
4-O-methylglucuronic acid content	Negative
Galactose content	Positive
Extractable glucose content (non crystalline)	Negative
Proportion of G lignin	Positive
Proportion of H lignin	Positive
Proportion of non-annotated phenolic compounds	Positive
Proportion of overall lignin	Positive
Ratio of cell wall carbohydrates to lignin	Positive
Fraction of wood (cross-sectional) area occupied by fibers	Positive
Average (cross-sectional) longest radial width of fibers	Negative
Average (cross-sectional) longest tangential width of fibers	Negative**
Average number of fibers per wood area	Negative
Average cross-sectional area of fibers	Negative

*TWG (total-wood glucose yield) relates the glucose released from saccharification after pretreatment to the estimated wood biomass per tree. In this way, TWG provides an estimate of the glucose yield from saccharification of all the wood from an entire tree. **This trait’s relationship to TWG is non-monotonic (i.e. the direction is not constant) over the full range of values and was therefore set to the direction of the relationship in the range of values encompassing the wild type, following usual conventions. In other words, traits marked with ** can be negatively correlated with TWG for a range of values and positively correlated with TWG for the rest, in which case the direction of the correlation around the wild-type value was reported here. This partly explains, for example, the apparent contradiction between the positive relationship for the proportion of S-lignin and TWG on the one hand and the “negative**” relationship of the S- to G-lignin ratio and TWG on the other hand.

The use of four individual models to construct either of the composite models enables predicting the four individual variables which compose TWG and TWGnp. While stem height, diameter and wood density are easily measured traits and therefore do not need to be predicted from wood biomass traits, the corresponding individual models identify wood anatomical and chemical traits that are associated with these three traits, thus providing useful information for feedstock improvement. Of great applied relevance, saccharification of glucose without and especially after a severe acidic pretreatment were predicted by individual models with good accuracy (Q^2^ = 0.70 and 0.49, respectively), based only on five and seven traits, respectively (Dataset S4).

As expected^14^, the individual models revealed that the ratio of S- to G-type lignin correlated positively with glucose release after pretreatment, but negatively with stem diameter (Dataset S4). Given that TWG integrates both stem diameter and glucose released by saccharification, the relationship between TWG and S- to G-lignin ratio was non-monotonic. In this case, the general influence of the S- to G-lignin ratio on the predicted TWG was determined by the direction of association in the range of values around the wild-type levels, which was negative (Table 1). This varying, albeit generally negative relationship between TWG and S- to G-lignin ratio in the composite model is consistent with the fact that the OPLS model displayed a small but overall negative relationship between TWG and the S- to G-lignin ratio (Fig. 3c). The relationship between lignin and TWG on the one hand, or saccharification on the other hand, will be discussed later in this article.

In addition to the S- to G-lignin ratio, three other traits that predicted glucose release after pretreatment were also associated with stem height and/or diameter (Dataset S4), demonstrating the interplay between biomass recalcitrance and biomass production. Of particular interest, low abundance cell wall monosaccharides such as rhamnose and arabinose were associated with both glucose release after acidic pretreatment and at least one of the biomass production traits - height, diameter and wood density (Dataset S4). While rhamnose was negatively associated with both biomass production and saccharification, and therefore with TWG (Table 1), arabinose had a non-linear relationship to saccharification and a negative impact on biomass production (Dataset S4), also resulting in a mainly negative impact on TWG (Table 1), consistent with the OPLS model. Hence, our modeling approach points towards quantitatively minor matrix polysaccharides as putative targets for selection or engineering of woody biomass in *Populus*.

In the composite models for TWG and TWGnp, the individual models for stem height, stem diameter and wood density were conserved (Dataset S4), which means that TWG and TWGnp only differ based on the individual models for glucose release. Most of the traits predicting saccharification of glucose were different between the pretreated and non-pretreated samples (Dataset S4). Furthermore, four out of the five traits predicting glucose release without pretreatment were related to cell wall chemical composition, while glucose release after pretreatment was associated with compositional and anatomical features in nearly equal parts (Dataset S4). Only one trait, rhamnose content, was associated with the prediction of both saccharification without and after pretreatment (Dataset 4). However, while rhamnose content was negatively associated with glucose release following pretreatment, it was positively associated with glucose release when no pretreatment was applied (Dataset S4). The relationship between TGWnp and rhamnose content was non-monotonic due the fact that rhamnose content is both positively associated with glucose release in the absence of pretreatment and negatively associated with stem diameter (Dataset S4). Hence, rhamnose content associated differently with the glucose yield from entire trees depending on pretreatment conditions, but the recurrence of rhamnose content among the identified predictive traits in both conditions exemplifies anyway the importance of such low abundant matrix component for total-wood glucose yield.

## Discussion

Our study identified putative diagnostic wood traits for the selection of trees with overall enhanced glucose yield, by taking into account biomass production and glucose release from enzymatic saccharification after a severe acidic pretreatment. Previous studies had started unravelling the links between saccharification and other wood properties by studying populations of natural variants^12–14^. The population of trees presented here was smaller and the trees were younger than in these previous studies^12–14^. Nevertheless, our work provided new information thanks to a different approach. We measured numerous traits from transgenic lines, which allowed us to analyze biological replicates and to generate combinations of traits which may not occur in nature. Furthermore, the estimated TWG enabled us to circumvent potential trade-offs between biomass production and recalcitrance. Examples exist in the literature of (genetically modified) trees with improved saccharification^18,20^ which is offset by a concomitant growth reduction^20^ or counter-acted by defects in xylem hydraulics^18,19,22^. Consequently, the use of the TWG calculation or of similar proxies that integrate biomass production and sugar release, in addition to traditionally monitored saccharification, may help future studies to identify superior trees.

Lignin content and composition are considered major determinants of biomass recalcitrance to saccharification, as verified in a large population of undomesticated *Populus trichocarpa* in which the S- to G-lignin ratio was positively correlated with glucose release after hydrothermal pretreatments^14^. Consistently, in our individual model for glucose release after acidic pretreatment the S- to G-lignin ratio was a positive contributor (Dataset S4), confirming the relationship between lignin composition and biomass recalcitrance. However, when considering TWG, which integrates biomass production and saccharification, both our OPLS model and composite model revealed an overall negative impact of the S- to G-lignin ratio (Fig. 3c, Table 1), likely because of its detrimental effect on stem diameter (Dataset S4). This observation interrogates the usefulness of increased S- to G-lignin ratio to improve the overall sugar yields in biochemical conversion of feedstocks.

In an earlier study, lignin content negatively correlated with saccharification in *Populus* trees with a ratio of S- to G-lignin below 2^14^, a range within which nearly all our trees fell (Fig. 1d). Unexpectedly, lignin content did not negatively correlate with glucose release after pretreatment in our PCA analysis (Fig. S1; Dataset S3) or in a pairwise comparison (Spearman’s rank correlation r_s_ = −0.09). Furthermore, lignin content did not contribute to predicting glucose release after pretreatment in the corresponding model (Dataset S4), indicating that lignin content did not greatly contribute to recalcitrance in our trees. Such discrepancy between the different studies on the effect of lignin content may be explained by differences in methods, age of the trees, genetic background, degree of domestication, and/or growth environment. Indeed, analysis of a set of *Populus trichocarpa* trees grown at two locations revealed different degrees of negative correlation between lignin content and glucose release depending on growing site^12^. On the other hand, these negative correlations between lignin content and saccharification were never statistically significant^12^. In addition, Studer *et al*.^14^ noted that a number of trees did not follow the general correlations between lignin content or composition and saccharification, leading them to propose that factors other than lignin can greatly influence biomass recalcitrance. Hence, the above observations are consistent with the emerging view that the woody biomass recalcitrance to saccharification is more complex than previously thought (for review see^11^), and that variations in lignin do not necessarily affect sugar release.

It is noteworthy that the traits predicting glucose release from saccharification greatly differed depending on whether the samples had undergone an acidic pretreatment or no pretreatment (Dataset S4). The pretreatment conditions have previously been shown to greatly affect saccharification yield for different feedstocks, as exemplified in *Populus* ^14^ and from systematic studies on wheat straw^24,25^. This suggests that the predictive traits that we identified for saccharification after pretreatment may be specifically related to the pretreatment condition that we applied. On the other hand, our acidic pretreatment, which aims at deconstructing hemicelluloses, displayed a high combined severity as revealed by the fact that there was on average over five times more xylose in the pretreatment liquid than what was released by saccharification from solid residues (Dataset S2). Such severe acidic pretreatment likely reflects the higher end of the range of pretreatment conditions, so that the pretreatment is not a limiting factor for saccharification, as might be desired also in industrial applications. Hence, our results likely reflect what occurs following an acidic pretreatment within a range of varying conditions that are relevant for industrial applications, but these observations cannot be generalized to all different types of pretreatments (e.g. alkali pretreatment or hydrothermal pretreatment).

An important source of variation between our lines may have been associated with tension wood (Fig. S2). Tension wood is regarded as a determinant of wood recalcitrance because it has been found to improve saccharification in willow, although at the expense of biomass production^26^. In the *Populus* genus, tension wood is associated with changes in cell wall monosaccharide composition such as decreases in xylose and mannose contents and concomitant increases in rhamnose, galacturonic acid and galactose contents^27,28^. Monosaccharide contents were also associated with TWG in our models (Table 1). The negative association of TWG with xylose and mannose contents together with the positive association of galactose content with TWG (Table 1) are consistent with an overall beneficial role of tension wood on TWG. However, the negative associations of rhamnose, non-crystalline glucose and arabinose contents with TWG (Fig. 3c, Table 1) cannot be explained by tension wood, suggesting that differences in pectin and hemicelluloses composition that are unrelated to tension wood also influence TWG. This observation is in line with studies in *Arabidopsis thaliana* ^29,30^ and *Populus* ^15,31^ suggesting hemicelluloses as a promising target for biotechnological engineering of biomass to increase saccharification without growth penalty.

It is interesting to note that among the matrix polysaccharides significantly associated with TWG (Fig. 3c, Table 1, Dataset S4), fucose, mannose, rhamnose and arabinose constitute quantitatively modest components of the wood biomass. Neither mannose nor fucose contributed to predicting saccharification but they negatively correlated with stem diameter and stem height, respectively (Dataset S4). Consequently, the composite model identified mannose as a negative contributor to TWG (Table 1) while fucose negatively correlated with TWG in both the composite model and the OPLS model (Fig. 3c, Table 1). Arabinose and rhamnose were associated with both saccharification and biomass production in the individual models constituting our composite model (Dataset S4) such that they had an overall negative association with TWG in both the composite model (Table 1) and the OPLS model (Fig. 3c). Hence, lower arabinose and rhamnose contents represent putative markers for a combination of increased biomass production and lower recalcitrance under acidic pretreatment conditions.

The individual models predicting saccharification without or after pretreatment represent the only differences between TWG and TWGnp because the models for predicting height, diameter and wood density remain the same. Hence, differences between TWG and TWGnp can be discussed in terms of differences between sugar release after or without pretreatment. Interestingly, glucose release without pretreatment was mainly associated with wood chemistry traits while glucose release after acidic pretreatment was associated with both features of wood chemistry and structure (Dataset S4), consistent with the fact that pretreatments are designed to act primarily on the chemistry of the biomass. Traits related to biomass structure may become important factors for saccharification after pretreatment, at least after acidic pretreatments as used in this study. Such an effect of especially acidic pretreatment on cell wall chemistry is also consistent with the observation that the predictive compositional traits differ to a large extent between pretreated and non-pretreated samples (Dataset S4).

A notable exception to the lack of overlap of predictive traits between pretreatment conditions is rhamnose content which associates with both, although in opposite directions (Dataset S4). Rhamnose content may therefore affect positively enzymatic saccharification in the context of non-pretreated wood biomass, while having a negative influence on acidic pretreatment and/or on the subsequent enzymatic saccharification in the context of the pretreated biomass. Rhamnose content is not only associated with saccharification but also negatively associated with stem diameter. The preponderance of rhamnose content in predicting wood biomass production as well as saccharification following two very different pretreatment conditions is somewhat surprising considering its low abundance in the cell walls. This result therefore exemplifies the importance of measuring quantitatively modest traits in future studies.

Our work relies on the use of transgenic lines designed to target specific genes, which allows us to discuss the potential genetic basis for the observed phenotypes. For instance, we found four *Populus* lines displaying significantly (p < 0.1) higher TWG than the wild type. While the causal link between the targeted genes and the improved TWG will require further investigation, three out of these four genes (in BI-2, BI-3 and BI-36) have not yet been characterized in relation to wood formation. This suggests that there probably remains a wealth of uncharacterized candidate genes which may provide markers for the selection of superior *Populus* trees or which represent targets for the biotechnological improvement of growth and biomass properties.

In conclusion, we uncovered a set of putative diagnostic traits for a combination of improved growth and biomass properties for saccharification after acidic pretreatment, which provides tentative tools for selecting *Populus* genotypes with high TWG. Indeed, *Populus* trees have been subject to domestication for a long time and there consequently exist numerous breeding populations^32–34^ from which promising individuals could be selected.

## Materials and Methods

Most of the data generated or analyzed during this study are included in this published article (and its Supplementary Information files). The rest of the raw data generated during and/or analyzed during the current study are available from the corresponding authors on reasonable request.

### Plant material and growth conditions

To create the BioImprove collection, transgenic hybrid aspen (*Populus tremula* x *tremuloides* Michx.) T89 clones were derived partially from a gene mining program performed at SweTree Technologies AB and partially from individual research groups at Umeå Plant Science Centre. The genes and the types of transgenic modifications are described in Dataset S1. Most of the lines in the BioImprove collection were hybrid aspen (*Populus tremula* x *tremuloides* Michx.) T89 clones that had been transformed by *Agrobacterium*-mediated gene transfer. Transformants were selected based on antibiotic resistance, grown and multiplied *in vitro* as previously described^35^. For each construct, three to five different lines were tested in an earlier study for wood chemistry^36^. From this study, we selected one transgenic line for each construct on the basis of giving the largest difference in wood chemistry. Fifty-one wild-type trees and four to five biological replicates for each transgenic line were grown for two months in previously described greenhouse conditions^17^. Each tree’s height, diameter (10 cm above ground) and mean internode length were measured, and 8-cm-long sections of stem were harvested 20 cm above ground. The bark was removed and the wood was freeze-dried and ground as previously described^17^ to perform cell wall chemistry and saccharification analyses. The cut trees were allowed to re-grow one new shoot, which was repeatedly trimmed at the height of 1 meter. After 10 months (i.e. a total age of the plants of 12 months), an 8-cm-long piece of the main stem 10 cm above ground was collected, debarked, dried, and used to monitor the anatomical and structural features of the wood.

### Cloning

The cloning procedure used to generate already published constructs (Dataset S1) has been described in the corresponding publications (Dataset S1). In addition, the antisense constructs for the lines BI-20 and BI-22 were generated using a similar procedure as described for BI-23^37^.

For down-regulation lines using RNAi (Dataset S1), a collection of previously identified^38^ hybrid aspen (*Populus tremula x tremuloides*) wood-expressed sequences (expressed sequence tags or ESTs) was used as a template to amplify the target sequences (as described in^39^). Gateway® cloning (Thermo Fisher Scientific, USA) was used to transfer each amplified sequence into the vector pK7GWiWG2(I)^39^, thus generating a construct for RNAi down-regulation of the target gene.

For overexpression lines (Dataset S1), mRNAs were isolated from both leaves and stems of hybrid aspen (*Populus tremula* x *tremuloides* Michx.) T89 clones and the corresponding cDNA were synthesized. The cDNA of the target genes for overexpression were amplified and introduced into the overexpression vector pK2GW7^40^ using Gateway® cloning (Thermo Fisher Scientific, USA).

### Cell wall compositional analyses

Relative contents of cell wall lignin and carbohydrates, as well as lignin composition, were measured by pyrolysis-gas chromatography/mass spectrometry and the data were processed as previously described^41^.

Cell wall monosaccharides were extracted by methanolysis with 2 M HCl/MeOH, derivatized by trimethylsilyl and measured as previously described^17^.

### Saccharification

As described previously^17^, wood samples were freeze-dried and roughly ground. From the resulting powder, the fraction encompassing particle sizes from 0.1 mm to 0.5 mm was collected for further processing. For each sample, 50 mg dry weight of substrate were submitted (or not) to an acidic pretreatment (1% (w/w) sulphuric acid) during 10 min at 165 °C using a single-mode microwave system (Initiator Exp, Biotage, Sweden). The resulting samples were centrifuged 15 min at 14,100 g in order to separate the solid fraction from the so-called pretreatment liquid. The solid fraction from pre-treated samples was washed with deionized water and with sodium citrate buffer (see details in^17^). Both pretreated and non-preteated samples were submitted to enzymatic hydrolysis 72 h at 45 °C under agitation, using a 1:1 (w/w) mixture of the liquid enzyme preparations Celluclast 1.5 L and Novozym 188 (Sigma-Aldrich). Celluclast 1.5 L had an activity of 74 FPU (Filter Paper Units)^42^ per g liquid enzyme preparation. Novozyme 188 had an activity of 15 β-glucosidase units (using 5 mM *p*-nitrophenyl glucopyranoside as substrate)^43^ per g liquid enzyme preparation. Reaction mixtures (total mass 1000 mg) contained 50 mg of untreated dry wood powder (or the solid residue obtained after pretreatment of 50 mg dry wood powder), the enzyme mixture (0.9 FPU and 0.18 β-glucosidase units), and sodium citrate buffer (pH 5.2, 0.05 M). The resulting liquid hydrolysates, as well as the above pretreatment liquid fractions, were analyzed using high-performance anion-exchange chromatography (HPAEC).

### Wood anatomical and structural features

SilviScan (CSIRO, Australia) measurements conducted at INNVENTIA/RISE were performed on all lines but three (BI-13, 21 and 26). Parallelepipedic radial pieces of wood were scanned with 2 mm increments as described previously^44–46^. The first measurement increment(s) covering not only wood but also the pith was (were) excluded from the analysis. Each remaining incremental measurement was weighted to reflect the total cross-sectional area that it represents in the wood. For each tree, the radial average was calculated for each trait measured by SilviScan (Dataset S2).

### Statistics and multivariate analyses

Average trait values of all the lines were compared by ANOVA. The lines were compared pairwise by post-ANOVA two-tailed Fisher’s tests while Spearman’s rank correlations allowed the comparison of traits across lines, both using Minitab 17 (Cleverbridge AG, Germany).

The PCA and the corresponding post-PCA OPLS (23) analyses were performed on all lines and all but 3 lines (BI-13, 21 and 26), respectively, using SIMCA 14.1 (MKS Data Analytics Solutions, Sweden). In the OLPS, traits related to saccharification were disregarded in our interpretation of TWG prediction because the effort intensive process of measuring saccharification, in addition to other traits, would allow direct calculation of TWG.

### Mathematical modeling

Models were created for stem height, stem diameter, wood density and glucose release without or after pre-treatment and 72 h enzymatic hydrolysis. To model these five traits based on wood biomass traits, these five traits were excluded from the set of traits used for modeling. In addition, the 18 remaining saccharification traits were also excluded from modeling for three reasons: (i) Our aim was to predict glucose yield from biomass properties which are not too difficult to measure so that they may already serve for application in the short term. (ii) In addition, measuring of the saccharification traits allows, for technical reasons, to measure the others at the same time. (iii) Finally, pairwise comparisons between saccharification traits suggested a lack of relationship between pre-treated and non-pretreated samples (compare e.g. Fig. 1e,f). Hence, glucose release would be measured at the same time as other saccharification traits, rendering its modeling superfluous.

Using R, numerous (≥30) models were generated with the aim of predicting each of the five traits used to calculate TWG (i.e. height, diameter, wood density and glucose release after pre-treatment). More precisely, for each of the five traits, three types of models were generated: (i) linear models which rely on linear relations between variables, (ii) Generalized Additive Models (GAMs^47,48^; package”mgcv”^49^) which allow combining linear terms and different types of non-linear terms whose relations to the dependent variable can be represented by smooth functions, (iii) Random forests^50^ (package”Ranger”^51^) which rely on numerous tree predictors each using random subsets of independent variables in order to allow comparing the trees to reach an optimal prediction and to evaluate how much each variable contributes to this prediction. Numerous models from each type were generated for each trait by iteratively modifying parameters such as the input independent variables and the criteria for fitting (e.g. number of trees in Random forests or gamma for GAMs). Finally, the predictivity of each model was evaluated by calculating their Q^2^, using a”leave-one-out” approach. For each trait, the model from each type with the highest Q^2^ value among its kind was selected (Dataset S4). Next, for each trait the type of model used *in fine* was also selected based on having the highest Q^2^ compared with the other types of models (Dataset S4). Finally, the ultimately selected models for each trait were combined into a composite model to predict TWG and this composite model was evaluated for goodness of fit (R^2^) and predictivity (Q^2^).

## Electronic supplementary material

Supplementary Information

Supplementary Dataset 1

Supplementary Dataset 2

Supplementary Dataset 3

Supplementary Dataset 4
