# Supplementary material for: A collection of genetically engineered Populus trees reveals wood biomass traits that predict glucose yield from enzymatic hydrolysis
Source: Sci Rep. 2017 Nov 17;7:15798. doi: 10.1038/s41598-017-16013-0 (PMC5693926; doi:10.1038/s41598-017-16013-0)
Supplement: Supplementary file 1 — Supplementary Information [file 41598_2017_16013_MOESM1_ESM.pdf]

# TITLE: A collection of genetically engineered *Populus* trees reveals wood biomass traits that predict glucose yield from enzymatic hydrolysis

Sacha Escamez[1,a], Madhavi Latha Gandla[2], Marta Derba-Maceluch[3], Sven-Olof Lundqvist[4], Ewa J. Mellerowicz[3], Leif J. Jönsson[2] and Hannele Tuominen[1,a]

[1]Department of Plant Physiology, Umeå University, Umeå Plant Science Centre (UPSC), SE-901 87 Umeå, Sweden.

[2]Department of Chemistry, Umeå University, SE-901 87 Umeå, Sweden.

[3]Department of Forest Genetics and Plant Physiology, Swedish University of Agricultural Sciences, Umeå Plant Science Centre (UPSC), SE-901 83 Umeå, Sweden.

[4]INNVENTIA AB, Box 5604, SE-114 86 Stockholm, Sweden.

**[a]Corresponding authors:**

Sacha Escamez

phone: +46907868545

e-mail: sacha.escamez@umu.se

Hannele Tuominen

phone: +46907869693

fax: +46907866676

e-mail: hannele.tuominen@umu.se

# Supplementary information

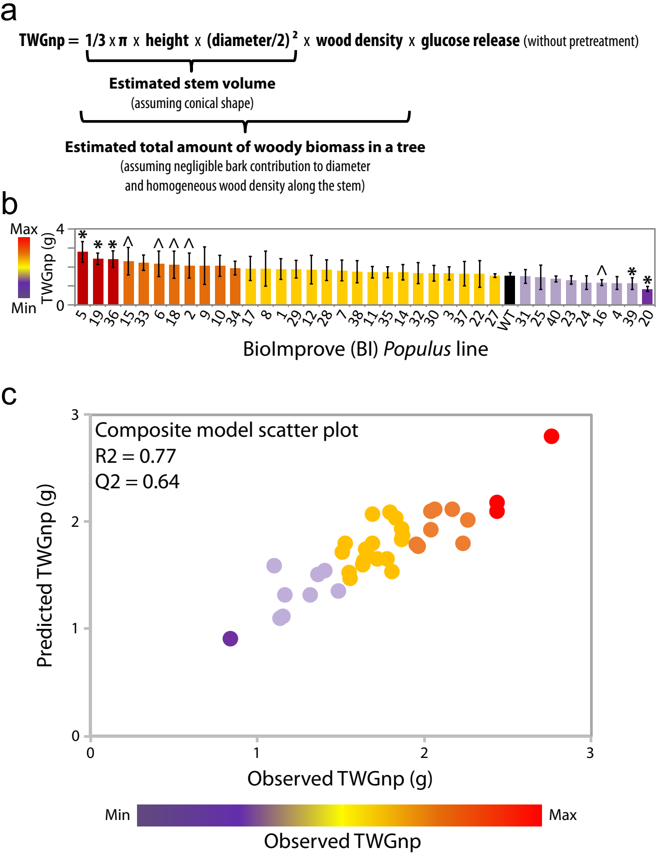


**Figure S1: TWG without pretreatment can be predicted by a specific subset of traits in a composite model**

(a) Formula for estimation of a tree's total-wood glucose yield (TWG) without pretreatment and 72h enzymatic hydrolysis, assuming conical shape, negligible bark contribution to diameter and homogeneous wood density.

(b) TWG of the BioImprove *Populus* lines (in g). Each histogram represents the average value for a transgenic *Populus* line (color) or wild-type (black). Error bars represent standard deviation. * and ^ indicate statiscally significant differences from wild-type (p<0,05 and p<0,1 respectively) following a post-ANOVA Fisher's test (n=3-5).

(c) Scatter plot showing for each *Populus* line (dots) the observed TWG without pretreatment(x-axis) versus the predicted TWG without pretreatment (y-axis). Q2 scores over 0.5 indicate significant predictivity of a model.

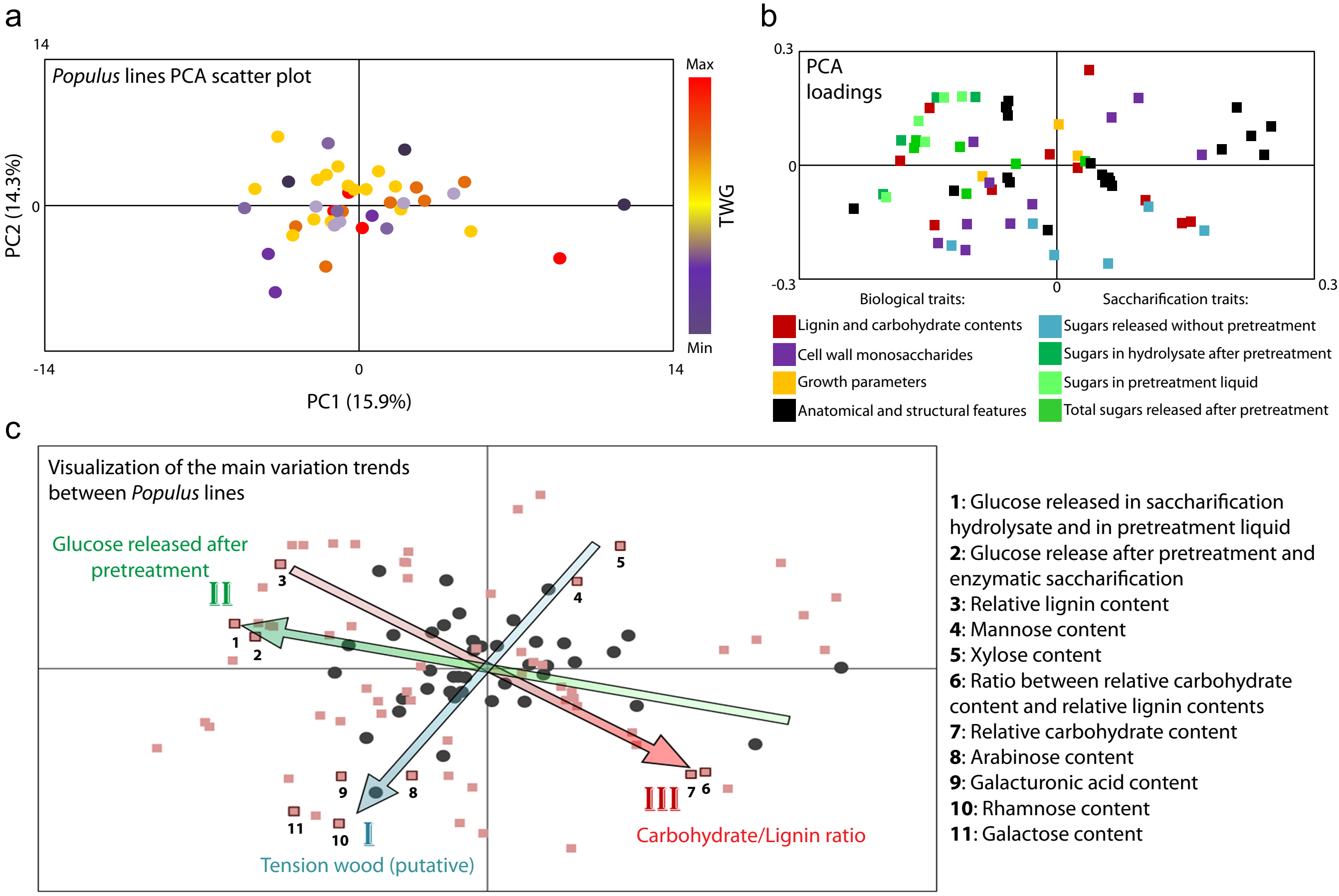


**Figure S2: Variation in biomass properties between *Populus* lines and apparent underlaying trends**

(a) Principal component analysis (PCA) scatter plot showing the position of each *Populus* line (dots) relative to the first (horizontal) and second (vertical) principal components (PC), explaining 15.9% and 14.3% of the variation in the dataset, respectively. The *Populus* lines were colored based on their total-wood glucose yield (TWG).

(b) Loadings from the PCA model used to analyze the variation between *Populus* lines (a). Each box represents one trait and is colored by category (Datasets S2,3).

(c) Graphic representation of the trends in biomass properties (important traits or combination of traits) apparently underlaying the variation between *Populus* lines. Although other traits can explain variation between the lines, three trends visually appear which can be interpreted as biologically meaningful or relevant for applications based on the current knowledge, and along which most of the lines separate. Each Roman numeral indicates such a major trend while the corresponding arrow indicates the direction of the trend: (I) Putative amount of reaction (tension) wood, revealed by the negative correlation between two groups of cell wall monosaccharides known to display such relation in the tension wood of hybrid aspen (ref25) and of other hard woods (ref26). (II) Glucose (and certain other monosaccharides) released after pretreatment. (III) Ratio betwen relative carbohydrate content (cellulose and hemicelluloses) and relative lignin content in the cell walls. Each dark grey dot represents a *Populus* line while each light-red box represents a loading (trait) in the PCA model. Numbers next to highlighted light-red boxes indicate which traits they correspond to in the right panel.
